# *Aelurostrongylus abstrusus* Antibody Seroprevalence Reveals That Cats Are at Risk of Infection throughout Germany

**DOI:** 10.3390/pathogens10081011

**Published:** 2021-08-10

**Authors:** Manuela Schnyder, Roland Schaper, Francesca Gori, Carola Hafner, Christina Strube

**Affiliations:** 1 Institute of Parasitology, Vetsuisse Faculty, University of Zurich, 8057 Zürich, Switzerland; francesca.gori@uzh.ch; 2 Independent Researcher, 51381 Leverkusen, Germany; roland.schaper@gmx.de; 3 IDEXX, Vet Med Labor GmbH, 70806 Kornwestheim, Germany; Carola-Hafner@idexx.com; 4 Institute for Parasitology, Centre for Infection Medicine, University of Veterinary Medicine Hannover, 30559 Hannover, Germany; christina.strube@tiho-hannover.de

**Keywords:** *Aelurostrongylus abstrusus*, feline lungworm, cat, Germany, antibody detection, ELISA, prevalence

## Abstract

Cats infected with the metastrongylid nematode *Aelurostrongylus abstrusus* may show clinical signs ranging from mild to severe respiratory disease or remain unobserved, despite damages present in the lung tissue. This study aimed to determine the seroprevalence and distribution of *A. abstrusus* in cats by testing serum samples from all over Germany to identify potential risk areas and strengthen disease awareness accordingly. Sera of 2998 cats were screened for the presence of antibodies against *A. abstrusus* by ELISA, and the data were evaluated by a geographic information system to visualise the regional distribution of the analysed samples. Overall, 12.0% of the samples tested positive (361/2998 cats, 95% confidence interval: 10.9–13.3%). Seropositive cats were identified throughout the country, suggesting that all cats in Germany with outdoor access are at risk of *A. abstrusus* infection and that the infection is overall underdiagnosed. Increased testing for *A. abstrusus* infection would allow earlier detection of infected animals, hence improving the life quality and health of cats and preventing potential death under anaesthesia.

## 1. Introduction

*Aelurostrongylus abstrusus* belongs to metastrongylid nematodes and is the most common feline lungworm worldwide being especially widespread across Europe [1]. Infected animals may show a range of clinical signs, from asymptomatic to severe respiratory distress. The most frequently observed signs are chronic cough, sneezing, nasal discharge, and dyspnea [2,3,4,5]. However, nonspecific signs or subclinical infections may also be observed [6,7], potentially leading to undiagnosed or misdiagnosed infections. Clinically apparent but asymptomatic infections with *A. abstrusus* should be considered seriously because lung tissue may be heavily affected by verminous pneumonia [8,9], even during prepatency [10]. Notably, such damages can lead to death during anaesthesia [11] and are therefore equally relevant in the frame of routine surgeries such as spaying and neutering.

The analysis of faecal samples by the Baermann technique is still the most frequently used diagnostic procedure to isolate and diagnose first-stage larvae (L1). This method relies on living L1 and, therefore, on fresh samples, as it is based on the principle of larval migration. Irregular or absent larval shedding, particularly in chronically and/or repeatedly infected cats, represents a further limitation [12,13,14].

Alternative diagnostic options have been described, including microscopic and cytologic examination of broncho-alveolar lavages [15], or, without anaesthesia, detecting parasite DNA from faecal or pharyngeal swab samples by PCR [16]. In addition, serological antibody detection was used recently for individual diagnosis and mass screening of cat populations [17,18,19]. However, *A. abstrusus* antibodies may not only be present before patency (i.e., 15 days post-infection) but also persist for weeks after successful anthelmintic treatment. In the latter case, antibody detection does not necessarily indicate a current infection [19]. Nevertheless, experimental and field data suggest a higher detection rate by serological procedures compared with copromicroscopic methods [1,17,18,20], which can be explained by earlier onset and more consistent persistence of antibodies than larval production.

In the study presented here, we performed serological mass-screening of cats in Germany by a validated ELISA [19] to detect *A. abstrusus* infections. Previous studies suggested potential cross-reactions with other lungworms such as *Troglostrongylus* sp. [17,21,22]. In Germany, however, the detection of lungworms other than *A. abstrusus* in cats is very rare, except for *Capillaria aerophila* (syn. *Eucoleus aerophilus*). Prevalence data for *A. abstrusus* in Germany based on copromicroscopic examinations range from 0.5% to 2.7%, and up to 6.6% in cats clinically suspected of *A. abstrusus* infection [23,24,25,26,27,28]. In contrast, *C. aerophila* was identified at a high prevalence (69.4%) in red foxes [29], but not in cats, except for a study where the prevalence of *Capillaria* eggs was 1.0%, although these could not be confirmed as *C. aerophila* eggs [26]. The latter species was identified in German wild cats (*Felis silvestris silvestris*), along with *Troglostrongylus brevior* and *Angiostrongylus* sp. [30], suggesting that these parasites occasionally occur in wild felids in the country.

This study aimed to determine the seroprevalence and potential geographic risk areas of *A. abstrusus* infection in cats by testing cat serum samples from all over the country to support targeted disease awareness and promote appropriate diagnostic procedures among veterinary practitioners.

## 2. Results

**Figure 1:**
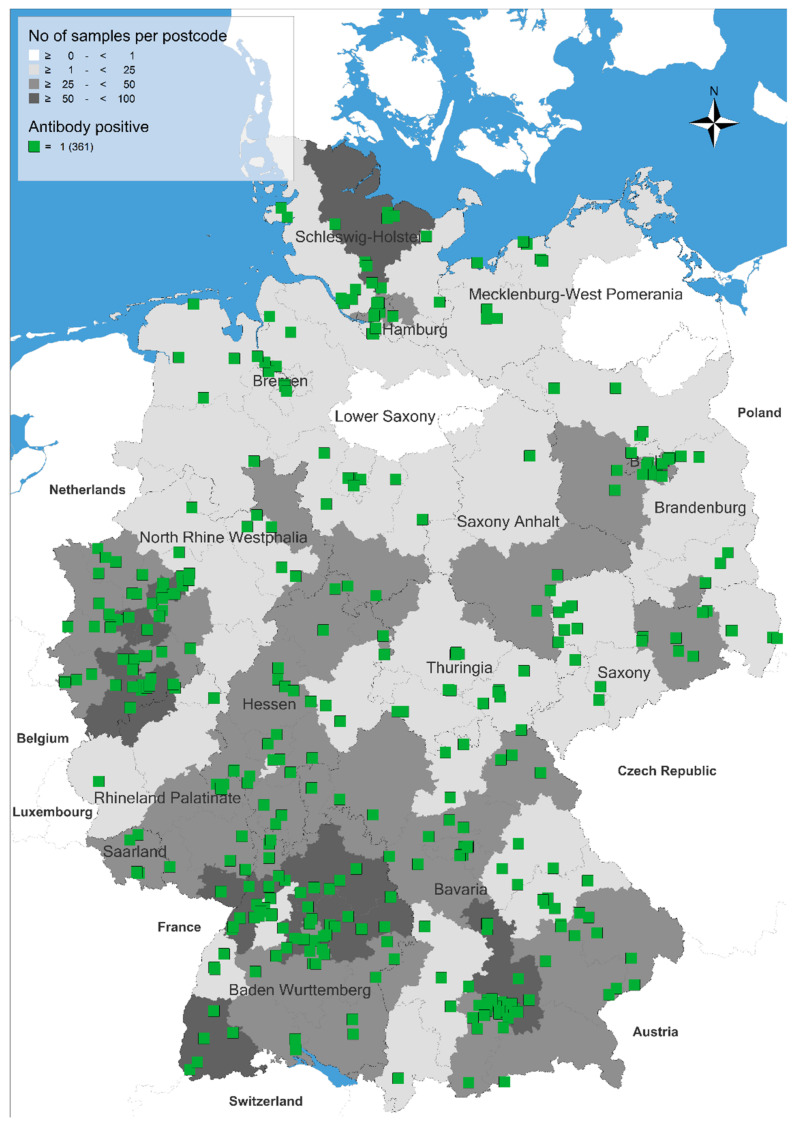
Occurrence of cats seropositive for antibodies against *Aelurostrongylus abstrusus* in Germany.

After analysing 2998 samples collected across Germany, an average seroprevalence of 12.0% (361/2998 cats, 95% confidence interval [CI]: 10.9–13.3%) of *A. abstrusus* was determined. Seropositive cats were identified throughout the country (Figure 1), and there was a clear correlation between the number of analysed cats and the number of positive cats, indicating that no area can be considered free of *A. abstrusus*.

A larger number of samples (>500) originated from Baden-Württemberg and Bavaria in southern Germany and North-Rhine Westphalia, the most western federal state of the country. In these regions, seroprevalences ranged from 9.0 to 14.8%, with an overlapping 95% CI (Table 1). Seroprevalences from other federal states ranged from 6.5% (Saxony-Anhalt) to 32.0% (Mecklenburg-West Pomerania), with large 95% CIs. Therefore, no statistical difference between the federal states was observed.

## 3. Discussion

With a seroprevalence of 12.0%, the rate of *A. abstrusus* antibody-positive cats in Germany is clearly higher compared with previous data obtained by copromicroscopic methods. Past studies, based on a similar selection of cat samples, where the Baermann method was applied, indicated a prevalence of 2.7% in 3167 cats analysed between 1999 and 2002 [23], of 0.5% in 8560 cats analysed between 2003 and 2010 [26], and of 1.0% in 837 stray and foster cats sampled between 2006 and 2007 [27]. By contrast, selecting cats with symptoms of respiratory disease, 5.6% of 231 and 6.6% of 391 cats were coproscopically confirmed *A. abstrusus* positive between 2003 and 2007 [25], and between 2009 and 2011.

Overall, these results are comparable to data from Switzerland, a country bordering the south of Germany: 10.7% of 4067 cats were seropositive for *A. abstrusus* antibodies [18], whereas between 0.8% and 2.3% of cats were coproscopically positive, with a higher prevalence in stray cats (6.5%) than owned cats (0.7%) [1,31]. Experimental studies showed that antibodies were detectable already 2–3 weeks before the onset of larval excretion [19], although isolated individuals may seroconvert later than 10 weeks post-infection [32]. Antibodies were then shown to persist as long as worms were present, therefore not necessarily correlating with patency [19]. Overall, a correlation between optical density (OD) values and the number of L1 per gram of faeces was observed, and it was concluded that low larval excretion may lead to OD values below the test threshold [32]. Antibodies also persisted for weeks after anthelmintic treatment [19,32]. By contrast, chronically infected cats or cats experiencing repeated infections (conceivable for all cats with unrestricted outdoor access) particularly showed an absence of larval shedding [7,13,33], hence precluding diagnosis by the Baermann method; this consequently leads to higher sensitivity and detection rates obtained with serological testing. The latter observations have also been made within highly endemic areas in Italy [17].

Our data indicate that the parasite is present in all German federal states. There were no significant differences between the seroprevalences of the different federal states; however, within single states, there are apparent areas with higher local endemicity, particularly where a high number of samples were analysed (North-Rhine Westphalia, Baden-Württemberg and Bavaria). Interestingly, these areas, together with the neighbouring state of Saarland, also showed higher prevalence for canine lungworms *Angiostrongylus vasorum* and *Crenosoma vulpis* [34], both sharing the same gastropod intermediate hosts with *A. abstrusus* [35]. Endemicity likely correlates with the presence of intermediate hosts and, consequently, with a suitable climate for coprophagic gastropods [18]. Different gastropod species are considered suitable intermediate hosts for *A. abstrusus*, and, among them, *Arion lusitanicus* is a very common slug in central Europe. Of note, 0.4% of 1587 analysed snails and slugs collected in four different sites in Germany were *A. abstrusus* positive [35]. Since diverging risk and protective factors were identified for different canine lungworms [34], additional factors influencing *A. abstrusus* occurrence are expected. Wetlands and a higher density of wooded areas are considered advantageous for *A. abstrusus* establishment, in contrast with an increased percentage of paved surfaces with a loss of natural soil function, negatively influencing its occurrence, but altogether regionally influencing the number of available intermediate hosts [18]. Although it may be presupposed that gastropods need a rather humid environment, epidemiological and geoclimatic studies showed that *A. abstrusus,* with its large temperature tolerance, can establish itself worldwide. On one hand, *A. abstrusus* L1 were shown to survive repeated freezing and thawing [36]. On the other hand, warmer temperature conditions (18–29 °C) allowed a higher rate of larval development in intermediate hosts [37], premising sufficient humidity.

In an earlier coproscopic study, positive cats were identified in the above-mentioned federal states and in northern Germany, but not in the centre of the country [28]. Therefore, increased *A. abstrusus* detection could therefore reflect either an increase of parasite occurrence over the years or be related to the low number of analysed samples between 2009–2011 [28]. Furthermore, considering the limited mobility of gastropods, additional support for establishment and dispersion is provided by potential interposed paratenic hosts such as rodents (i.e., *Apodemus agrarius*) [38] or reptiles, amphibians, and birds [39], probably feeding more frequently on gastropods than cats themselves. Therefore, it can be summarised that all cats with outdoor access may be at risk of infection in Germany, and in any *A. abstrusus* endemic area, provided that the geoclimatic factors are favourable.

Although serology cannot discriminate current infections from recently dewormed animals, the ELISA employed in the present work has the advantage of identifying positive cats with few or even absent clinical signs and, partially, before patency [18]. Compared with serology, a further disadvantage of L1 isolation from faecal samples is that it may be challenging to obtain such samples from cats, especially from free-ranging animals at high risk of infection. Further diagnostic tools may be used, reviewed in [40,41]. Diagnostic imaging, i.e., radiology and computed tomography, illustrates the onset of such damages despite the absence of clinical signs [8,32]. Infected cats may remain undetected by the animal owner for some time, or the clinical signs may be attributed to another respiratory disease, such as feline asthma, pulmonary oedema, neoplasia, or pneumonia-causing pathogens [2,42]. In both cases, the additional risk for lethal outcome exists if cats are subjected to anaesthesia: in 9% of cats whose death was associated with anaesthesia, lung tissue damages were caused by *A. abstrusus* [11]. Identifying infected cats by serology is therefore useful and relevant in symptomatic and asymptomatic animals, especially in view of the lung tissue damages induced by the parasite.

This risk is highly relevant considering that 12% of cats in Germany showed antibodies against *A. abstrusus* and that cats are frequently neutered, treated for abscesses or anesthetized for other reasons. Seroprevalences were actually almost twice as high in cats of some investigated Mediterranean areas, including Greece (27.3%) and Italy (21.4%, 22.5%) [17,21,22]. Several factors influencing the risk of an individual cat becoming infected with *A. abstrusus*, partially contradictory, were described [1,3,43,44]. In summary, less well-cared-for cats with high roaming and hunting activity are at higher risk, directly or indirectly influenced by their neutering status and age [18]. In addition, it cannot be excluded that other infrequently occurring metastrongylid lungworms (*Troglostrongylus* sp. and *Angiostrongylus* sp.) identified in wild cats of Germany [30] may also infect domestic cats in the future, as observed in countries with higher endemicity. In Italy, a spillover from wild cats to domestic cats was observed for *T. brevior* [2], while *Angiostrongylus chabaudi* or *Angiostrongylus vasorum* is still very seldom found in domestic cats [45,46,47]. They all belong to the same taxonomic family, have a comparable life cycle, and may result in serological cross-reactions when testing for anti-*A. abstrusus* antibodies [17,21]. *Capillaria* spp. instead belong to the trichurids and are, thus, taxonomically distant from the metastrongylids and not expected to cross-react with them [19]. Therefore, serological detection of antibodies in German cats is currently expected to indicate *A. abstrusus* infection.

A fundamental advantage of this study is the use of serology, allowing efficient mass screening of cats; this is particularly valuable in the case of cats with outdoor access, where cats must be confined for the collection of faecal samples and Baermann analysis. Thus, it is easier for veterinary practitioners to collect a blood sample in case of clinical suspicion. A limiting factor of the study is the lack of information regarding anamnestic data and clinical signs of the investigated cats. However, advanced clinical signs often go undetected, and our data confirm that the infection is underestimated overall. This work highlights the benefit of increased testing for *A. abstrusus* infections in cats, with the goal of increasing disease awareness for a more accurate and earlier identification of infected animals prone to reduced quality of life and even death upon anaesthesia.

## 4. Materials and Methods

### 4.1. Cat Sera

Sera of 2998 cats from all over Germany submitted by veterinarians for haematological or clinical chemistry analyses and various other medical reasons were collected between November 2018 and March 2019. The sera were provided by IDEXX and sent to the Institute of Parasitology, University of Zurich in Switzerland.

### 4.2. ELISA

Serum samples were stored at −20 °C and thawed before being tested for antibodies against *A. abstrusus* by a previously described and validated indirect ELISA (sensitivity 88.2%, specificity 90.0%) [19]. The ELISA was performed with modifications: we used Nunc Immobilizer Amino Plates (Thermo Fisher Scientific, Roskilde, Denmark) coated with recombinant *Dictyocaulus viviparus* major sperm protein (MSP, 0.250 µg/well), serum diluted 1:200, and a goat anti-feline IgG peroxidase-labelled conjugate (Southern Biotech, Birmingham, AL, USA) at a dilution of 1:9000. The absorbance values were read in a Multiscan ELISA reader (Tecan Infinite F50, Hombrechtikon, Switzerland), initially at 450 nm to determine the exact and optimal time to stop the reaction with sulphuric acid. Subsequently, the plate was read at 492 nm. Each plate was run with a substrate control, two positive controls (sera from experimentally infected cats), two negative controls (uninfected laboratory cats) and a conjugate control. A reference serum was added twice to each plate to calculate a correction factor for adjustment between plates [48]. Test threshold (0.295) was determined with 300 randomly selected samples based on the mean value of optical density plus three standard deviations [49].

### 4.3. Mapping, Statistical Analyses

The collected data were analysed by a geographic information system (GIS) using the program RegioGraph 10 (GfK GeoMarketing, Bruchsal, Germany) to visualise the regional distribution of collected and analysed serum samples and *A. abstrusus* antibody-positive samples. Based on four digits as points of reference, the locations of positive samples were displayed on maps with administrative and postcode boundaries.

Excel 2016 for Windows (Microsoft Corporation, Redmond, WA, USA) was used to calculate the prevalence values, the 95% CI and for calculating means and standard deviations (SD).

## Figures and Tables

**Table 1:** Number of serologically examined seropositive cats in federal states of Germany.

Federal State of Germany	Number of Examined Sera (n)	Number of Positive Sera (n)	Seroprevalence and 95% Confidence Intervals (%, Range)
Baden-Württemberg	546	64	11.7 (9.1–14.7)
Bavaria	548	77	14.1 (11.3–17.2)
Berlin	105	9	8.3 (4.0–15.6)
Brandenburg	110	14	12.7 (7.1–20.4)
Bremen	17	4	23.5 (6.8–49.9)
Hamburg	78	8	10.3 (4.5–19.2)
Hesse	252	23	9.1 (5.9–13.4)
Mecklenburg-West Pomerania	25	8	32.0 (14.9–53.5)
Lower Saxony	186	20	10.8 (6.7–16.1)
North-Rhine Westphalia	587	61	10.4 (8.0–13.1)
Rhineland Palatinate	133	12	9.0 (4.7–15.2)
Saarland	37	5	13.5 (4.5–28.8)
Saxony	138	19	13.8 (8.5–20.7)
Saxony-Anhalt	62	4	6.5 (1.8–15.7)
Schleswig-Holstein	122	19	15.6 (9.6–23.2)
Thuringia	52	14	26.9 (15.6–41.0)
Total	2998	361	12.0 (10.9–13.3)

## Data Availability

Data supporting reported results is contained within the article.
